# Symmetric molecules with 1,4-triazole moieties as potent inhibitors of tumour-associated lactate dehydrogenase-A

**DOI:** 10.1080/14756366.2017.1404593

**Published:** 2017-12-04

**Authors:** Abdul-Malek S. Altamimi, Ahmed M. Alafeefy, Agnese Balode, Igor Vozny, Aleksandrs Pustenko, Mohey Eldin El Shikh, Fatmah A. S. Alasmary, Sherif A. Abdel-Gawad, Raivis Žalubovskis

**Affiliations:** a Department of Pharmaceutical Chemistry, College of Pharmacy, Prince Sattam Bin Abdulaziz University, Alkharj, Saudi Arabia;; b Department of Chemistry, Kulliyyah of Science, International Islamic University Malaysia;; c Latvian Institute of Organic Synthesis, Riga, Latvia;; d Institute of Technology of Organic Chemistry, Faculty of Materials Science and Applied Chemistry, Riga Technical University, Riga, Latvia;; e Experimental Medicine and Rheumatology, William Harvey Research Institute, Queen Mary University of London, London, UK;; f Chemistry Department, College of Science, King Saud University, Saudi Arabia, Riyadh;; g Analytical Chemistry Department, Faculty of Pharmacy, Cairo University, Cairo, Egypt

**Keywords:** Lactate dehydrogenase, triazole, inhibitors

## Abstract

A series of symmetric molecules incorporating aryl or pyridyl moieties as central core and 1,4-substituted triazoles as a side bridge was synthesised. The new compounds were investigated as lactate dehydro-genase (LDH, EC 1.1.1.27) inhibitors. The cancer associated LDHA isoform was inhibited with IC_50_ = 117–174 µM. Seven compounds exhibited better LDHA inhibition (IC_50_ 117–136 µM) compared to known LDH inhibitor – galloflavin (IC_50_ 157 µM).

## Introduction

The lactate dehydrogenase (LDH, EC 1.1.1.27) is one of the most abundant proteins and it is expressed in all tissues^1^. The main function of LDH is interconversion of lactate and pyruvate with accompanying interconversion of NAD^+^ and NADH.

Three LDH isoforms are present in humans – LDHA, LDHB and LDHC. LDHA and LDHB are expressed in all cells, whereas LDHC is produced only in testis^1^. In the active form, LDH is a tetramer formed of LDHA and LDHB in various ratios making five tetramers: LDH1 (4 × LDHB), LDH2 (1 × LDHA/3 × LDHB), LDH3 (2 × LDHA/2 × LDHB), LDH4 (1 × LDHA/3 × LDHB) and LDH5 (4 × LDHA)^2^.

**Figure 1.**
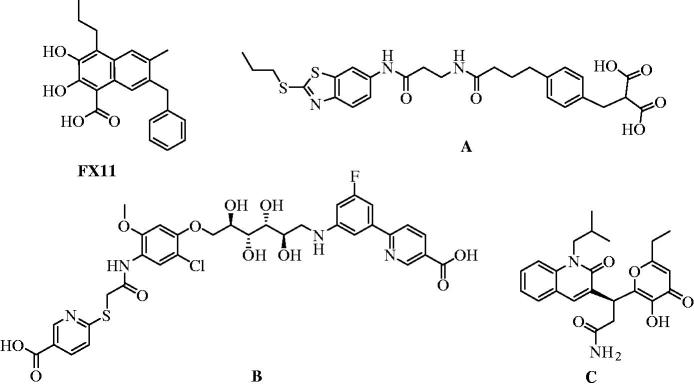
Examples of chemical structures of known LDHA inhibitors.

In normal cells, predominant is LDHB where it converts lactate to pyruvate with interconversion of NAD^+^ into NADH, which allows cells to use lactate as a nutrient source for oxidative metabolism, and/or for gluconeogenesis^2^. LDHA is the predominant isoform found in skeletal muscle and other highly glycolytic tissues. In contrast to LDHB, LDHA has a higher affinity for pyruvate, that is, LDHA and LDH5 tetramer in particular predominantly converts pyruvate to lactate with consumption of one NADH molecule to produce NAD^+^ which in turn is essential in glycolysis^3^. Cancer cells mainly generate energy through glycolysis even in the presence of normal oxygen pressure^4^. Since the LDHA is the final enzyme in glycolysis pathway where generated NAD^+^ is necessary for continued high glycolysis rate in cancer cells (Warburg effect)^4^, LDHA is an important supporter of glucose metabolism in cancer cells and can affect tumourigenesis and metastasis^5^. Additionally, elevated levels of LDHA are markers of many tumours, the majority of them are highly glycolytic, and high LDHA levels are related with poor prognosis, for instance in several human malignancies^6^. Therefore, LDHA is defined as anticancer drug target^6^^,^^7^. Notably, a limited number of LDHA inhibitors is reported in the literature so far^8^. In several papers, very promising LDHA inhibition results have been reported. As a one of the first inhibitors with low-micromolar inhibition activity (*K*_i_ = 13.5 μM) compound **FX11** (Figure 1) along with two similar compounds was reported in 2010^9^. Utilising NMR and SPR (surface plasmon resonance) fragment based hit identification technique and further optimisation of structure a series with low- and submicromolar LDHA inhibition was obtained with best lead **A** (IC_50_ = 0.27 μM)^10^. In the other study, also using fragment-based hit identification, series of new LDHA inhibitors was obtained. In this series, IC_50_ values ranged from 59 to 0.12 μM, where the best inhibition was observed for compound **B**.^11^ It was noted that in this series carbohydrate moiety in the middle of the molecule and its stereochemistry had significant influence on the inhibition activity. In very recent work through docking-based virtual screening, several potential LDHA inhibitors were identified. One of the compounds identified (**C**) showed very good LDHA inhibition potency *in vitro* with an IC_50_ value of 0.33 μM.^12^

## Results and discussions

Here, we report the synthesis of symmetric molecules incorporating aryl or pyridyl moieties as central core and 1,4-substituted triazoles as a side bridge and they evaluation as LDHA inhibitors.

At the beginning of our study based on literature data^11^, we assumed that V-shape structures are beneficiary for good LDHA inhibition. Our assumption was based on published X-ray structures, for instance for **B**–LDHA complex (PDB code 4I9H). It was also obvious that besides V-shape of the molecule and appropriate length of V-“arms” the terminal groups have to be able to make hydrogen bonding, therefore we chose carboxylic, sulphonamide and nitro groups as terminal ones for this study.

### Chemistry

**Scheme 1.**
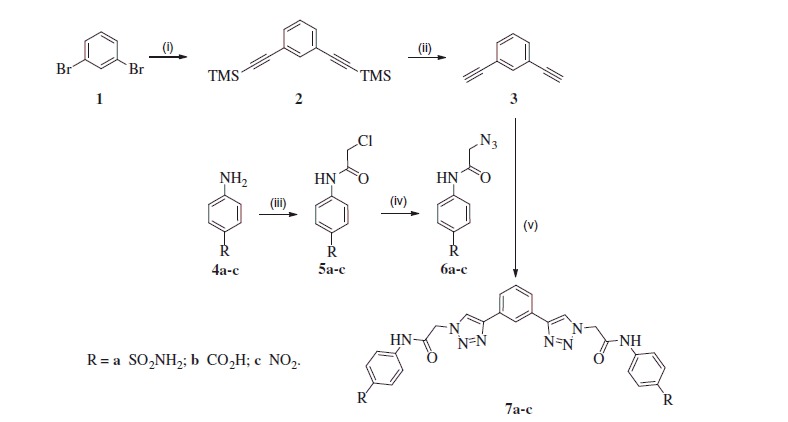
Reagents and conditions: (i) trimethylsilylacetylene, Pd_2_(PPh_3_)_2_, CuI, *i-*Pr_2_NH, THF, 70 °C; (ii) KF, MeOH/THF, rt, 69% for two steps; (iii) chloroacetyl chloride, K_2_CO_3_, THF, 0 °C, **5a** (98%), **5b** (84%), **5c** (89%); (iv) NaN_3_, DMF, rt, **6a** (97%), **6b** (68%), **6c** (95%); (v) CuSO_4_^.^5H_2_O, sodium ascorbate, AcOH, DMF/H_2_O, rt, **7a** (32%), **7b** (71%), **7c** (41%). All detailed experimental procedures are provided in the Supplemental data.

The synthesis of desired inhibitors **7a-c** was started from commercially available 1,3-dibromobenzene (**1**) which was reacted with trimethylsilylacetylene in Sonogashira reaction to provide bis-TMS protected derivative **2** (Scheme 1). Following deprotection with KF afforded building block **3** in good yield over two steps^13^. Azides **6a-c** necessary for Cu-mediated click reaction were prepared in two steps from commercially available anilines **4a-c**. Acylation of anilines **4a-c** with chloroacetyl chloride afforded chlorides **5a-c**^14–16^ in good yields and following treatment with NaN_3_ provided azide building blocks **6a-c** also in good yields. Reaction of building block **3** with **6a-c** under acidic click reaction condition^17^ provided inhibitors **7a-c**.

**Scheme 2.**
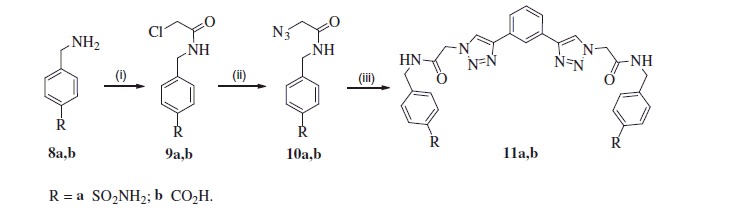
Reagents and conditions: (i) chloroacetyl chloride, K_2_CO_3_, THF, 0 °C to rt, **9a** (33%), **9b** (69%); (ii) NaN_3_, DMF, rt, **10a** (78%), **10b** (77); (iii) **3**, CuSO_4_^.^5H_2_O, sodium ascorbate, AcOH, DMF/H_2_O, rt, **11a** (17%), **11b** (59%).

The same strategy as described earlier was utilised for the synthesis of inhibitors **11a** and **11b**. First, aminomethylphenyl derivatives **8a,b** were acylated with chloroacelyl chloride to obtain intermediates **9a** and **9b**, which were converted into corresponding azides **10a,b** by treatment with NaN_3_ (Scheme 2). Following reaction of azides **10a,b** with building block **3** under acidic click reaction conditions provided desired inhibitors **11a** and **11b**.

**Scheme 3.**
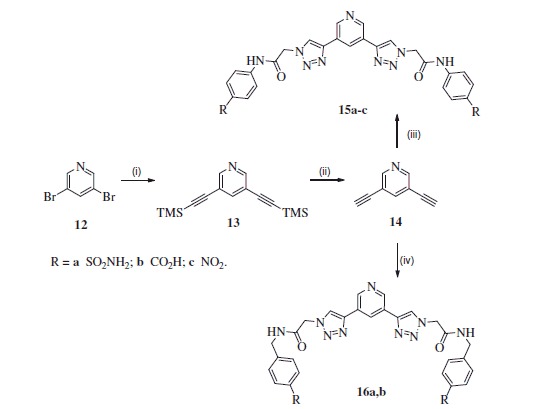
Reagents and conditions: (i) trimethylsilylacetylene, Pd_2_(PPh_3_)_2_, CuI, Et_3_N, 75 °C; (ii) K_2_CO_3_, MeOH/THF, rt, 67% for two steps; (iii) **6a-c**, CuSO_4_^.^5H_2_O, sodium ascorbate, AcOH, DMF/H_2_O, rt, **15a** (85%), **15b** (39%), **15c** (73%); (iv) **10a,b**, CuSO_4_^.^5H_2_O, sodium ascorbate, AcOH, DMF/H_2_O, rt, **16a** (63%), **16b** (46%).

Bis-acetylenylpyridine building block **14** was prepared in similar way to compound **3**, where 3,5-dibromopyridine **12** was first reacted with TMS-acetylene under Sonogashira reaction conditions and then TMS were eliminated in obtained intermediate **13** by treatment with K_2_CO_3_ proving building block **14** in 67% yield over two steps (Scheme 3)^18^.

The reaction between building blocks **13** and **6a-c** under acidic click reaction conditions provided inhibitors **15a-c** (Scheme 3). In turn, treatment of **13** by azides **10a,b** under the same condition provided inhibitors **16a**,**b** with extended bridge.

**Scheme 4.**
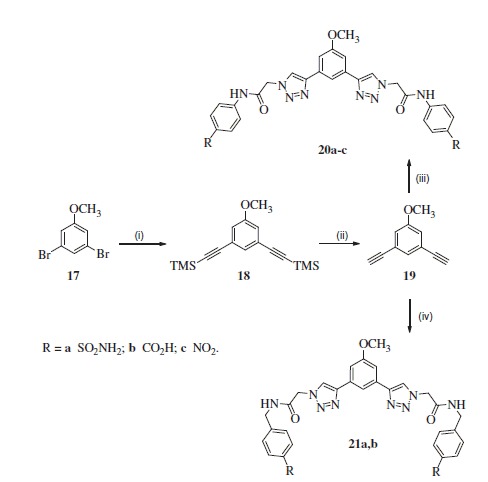
Reagents and conditions: (i) trimethylsilylacetylene, Pd_2_(PPh_3_)_2_, CuI, Et_3_N, THF 65 °C; (ii) KOH, MeOH/THF, rt, 61% for two steps; (iii) **6a-c**, CuSO_4_^.^5H_2_O, sodium ascorbate, AcOH, DMF/H_2_O, rt, **20a** (64%), **20b** (56%), **20c** (47%); (iv) **10a,b**, CuSO_4_^.^5H_2_O, sodium ascorbate, AcOH, DMF/H_2_O, rt, **21a** (31%), **21b** (85%).

And finally, for the synthesis of inhibitors **20a-c** and **21a,b,** anisole building block **19** was synthesised in analogy to **3** and **14** as above, starting synthesis from dibromoanisole **17**. Bis-TMS protected intermediate **18** was obtained under Sonogashira reaction conditions and following deprotection by potassium hydroxide provided building block **19** in 61% yield over two steps (Scheme 4)^19^.

Treatment of **19** with azides **6a-c** under the same condition as described above provided inhibitors **20a-c** (Scheme 4). In turn, treatment of **19** with azides **10a,b** afforded desired inhibitors **21a,b**.

The structures of all new compounds synthesised were fully approved by ^1^H and ^13^C NMR, IR and HRMS data (see Supplemental data).

### Inhibition studies

All 15 symmetrical compounds were evaluated for their ability to inhibit LDHA, for the comparison galloflavin as a known isoform nonselective LDHA inhibitor^20^ was used. Our choice of galloflavin was based on the fact that it is extensively studied as potent anticancer agents in recent years^21–25^; additionally, galloflavin is already commercially available.

Even though all compounds exhibited similar LDHA inhibition and it is difficult to perform structure-activity-relationship (SAR) analysis, we can divide this compound into two groups. First one, the most active compounds (**7a**, **7b**, **15a**, **15b**, **16b**, **20b** and **21b**) showed better LDHA inhibition compared to galloflalvin, ranging IC_50_ values from 117 to 136 µM for compounds in first group, whereas galloflavin has IC_50_ = 157 µM (Table 1). The most active compound in this group was **16b** (IC_50_ = 117 µM), which incorporated pyridyl moiety as a central core and carboxylic groups as a terminal ones. Second group of compounds (**7c**, **11a**, **11b**, **15c**, **16a**, **20a**, **20c** and **21a**) exhibited equal or weaker inhibition compared to galloflavin, ranging IC_50_ values from 156 to 174 µM. The most active compounds in this group were **11a**, **11b** and **15c** (IC_50_ 158, 156 and 156 µM, respectively). Similar activity of this three compounds is hard to explain, where compounds **11a** and **11b** have phenyl ring as a central core and sulphonamide and carboxyl groups as terminal ones on aminomethylphenyl moieties, but compounds **15c** has pyridyl central moiety and nitro groups as terminal one on shorter, aniline containing, bridge.

**Table 1.** Inhibition data of human LDHA.

Compound	Inhibition, IC_50_ (μM)*
**7a**	128
**7b**	120
**7c**	172
**11a**	158
**11b**	156
**15a**	136
**15b**	125
**15c**	156
**16a**	174
**16b**	117
**20a**	165
**20b**	127
**20c**	163
**21a**	173
**21b**	128
Galloflavin	157
Blank	0

* Mean from three different assays, by colorimetric assay measuring the absorbance at 450 nm (errors were in the range of ±5–10% of the reported values).

We hypothesise that the putative interaction of the inhibitors described in this work with LDHA is similar to the published one, that is, inhibitors bound in the active centre close to conserved residues involved in the catalytic processing of LDHA substrates^26^. We also assume that at the same time the NADH cofactor is bound in the active centre near the inhibitor molecule. This binding region of the protein is known to undergo considerable conformational changes during the catalytic cycle, that is, it has rather high flexibility. Therefore, inhibitor binding might be unstable and this reflects in IC_50_ values obtained.

## Conclusions

In conclusion, a series of new symmetric molecules have been designed and synthesises as potent LDHA inhibitors. The compounds synthesised exhibited promising *in vitro* LDHA inhibition activity, where seven compounds were better inhibitors (IC_50_ 117–136 µM) as known LDH inhibitor – galloflavin (IC_50_ 157 µM), and other eight showed equal or slightly lower inhibitory activity (IC_50_ 156–174 µM) as galloflavin. The results obtained are promising base for further development of novel LDH inhibitors.

## Supplementary Material

IENZ_1404593_Supplementary_Material.pdf
