# Supplementary material for: Symmetric molecules with 1,4-triazole moieties as potent inhibitors of tumour-associated lactate dehydrogenase-A
Source: J Enzyme Inhib Med Chem. 2017 Dec 4;33(1):147–50. doi: 10.1080/14756366.2017.1404593 (PMC6009863; doi:10.1080/14756366.2017.1404593)
Supplement: IENZ_1404593_Supplementary_Material.pdf [file IENZ_A_1404593_SM9579.pdf]

Symmetric molecules with 1,4-triazole moieties as potent inhibitors of tumor associated lactate dehydrogenase-A

Abdul-Malek S. Altamimi[a], Ahmed M. Alafeefy[b]*, Agnese Balode[c,d], Igor Vozny[c], Aleksandrs Pustenko[c,d], Mohey Eldin El Shikh[e], Fatmah A. S. Alasmary[f], Sherif A.Abdel-Gawad[a,g], Raivis Žalubovskis[c]*

[a] Department of Pharmaceutical Chemistry, College of Pharmacy, Prince Sattam Bin Abdulaziz University, P.O. Box 173, Alkharj, 11942, Saudi Arabia

[b] Department of Chemistry, Kulliyyah of Science, International Islamic University Malaysia

[c] Latvian Institute of Organic Synthesis, Aizkraukles 21, LV-1006, Riga, Latvia

[d] Institute of Technology of Organic Chemistry, Faculty of Materials Science and Applied Chemistry, Riga Technical University, Paula Valdena Str. 3/7, Riga LV-1048, Latvia

[e] Experimental Medicine and Rheumatology, William Harvey Research Institute, Queen Mary University of London, Charterhouse Square, London, EC1M 6BQ, UK

[f] Chemistry Department, College of Science, King Saud University, Riyadh 11362, Saudi Arabia.

[g] Analytical Chemistry Department, Faculty of Pharmacy, Cairo University, Cairo, ET-11562, Egypt

Supporting Information

Contents

1. General information……………………………………………………2
2. Reaction procedures……………………………………………………2
3. LDHA inhibition assay...18
4. References……………………………………………………………..18

1. General information

Chemistry

Reagents and starting materials were obtained from commercial sources and used as received. Flash chromatography was carried out using Merck silica gel (230-400 mesh). Thin-layer chromatography was performed on silica gel, spots were visualized with UV light (254 and 365 nm). Melting points were determined on an OptiMelt automated melting point system. IR spectra were measured on Shimadzu FTIR IR Prestige-21 spectrometer. NMR spectra were recorded on Bruker (400 MHz) spectrometer with chemical shifts values (d) in ppm relative to TMS using the residual DMSO-d_6 signal (^{1}H 2.50; ^{13}C 39.52) or $CDCl_3$ signal (^{1}H 7.26; ^{13}C 77.16) as an internal standard. HRMS data were obtained with a Q-TOF micro high resolution mass spectrometer with ESI (ESI^+/ESI).

Reaction procedures

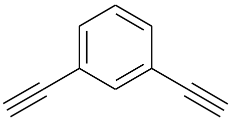

1,3-Diethynylbenzene (3)[1]

A solution of 1,3-dibromobenzene (**1**) (2.00 g, 8.55 mmol), *i*-Pr_2NH (6.86 g, 67.8 mmol) in dry THF (20 mL) was degassed with argon for 10 min. $Pd(PPh_3)_2Cl_2$ (0.59 g, 0.85 mmol), CuI (0.16 g, 0.85 mmol) and trimethylsilylacetylene (3.33 g, 33.9 mmol) were added and stirred under Ar at 70 °C overnight. After cooling to room temperature, the reaction mixture was filtered through a pad of celite and concentrated in vacuum. The crude product was purified by column chromatography on silica gel (hexane 100%). Obtained 1,3-bis((trimethylsilyl)-ethynyl)benzene (**2**) was dissolved in a mixture of MeOH (60 mL) and THF (30 mL). KF (3.72 g, 63.9 mmol) was added and stirred at room temperature for 1h. After evaporating the solvent, the mixture was dissolved in EtOAc (30 mL) and washed with water (3 × 30 mL). The organic phase was dried over Na_2SO_4 and concentrated in vacuum to provide 1,3-diethynylbenzene (**3**) as yellow oil (0.81 g, 69%).

^{1}H NMR (400 MHz, DMSO-d_6) δ: 4.27 (s, 2H), 7.38–7.43 (m, 1H), 7.49–7.56 (m, 3H).

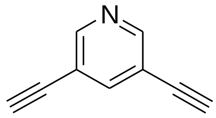

1,3-Diethynylpyridine (14)[2]

A sealed tube was charged with 3,5-dibromopyridine (**12**) (2.00 g, 8.44 mmol), $Pd_2(dba)_3$ (0.19 g, 0.21 mmol), CuI (0.06 g, 0.34 mmol), PPh_3 (0.44 g, 1.69 mmol), Et_3N (50 mL) and trimethylsilylacetylene (3.00 mL, 21.11 mmol). The mixture was stirred under Ar at 75 °C for 18 h. After cooling to room temperature the reaction mixture was filtered through celite pad and concentrated in vacuum. The crude product was purified by column chromatography on silica gel (hexane/EtOAc 15:1) to provide bis-TMS-protected intermediate **13** in quantitative yield. Compound **13** was dissolved in Et_2O/MeOH (1:2) and K_2CO_3 (6.51 g, 47.1 mmol) was added. The reaction mixture was stirred for the 1 h at room temperature, diluted with Et_2O and washed with H_2O (3×75 mL). The organic layer was dried over Na_2SO_4 and concentrated in vacuum to dryness to give 1,3-diethynylpyridine (0.72 g, 67%) as light brown solid. Mp 69–70 °C.

^{1}H NMR (400 MHz, DMSO-d_6) δ: 4.52 (s, 2H), 8.02 (t, J = 2.0 Hz, 1H), 8.67 (d, J = 2.0 Hz, 2H).

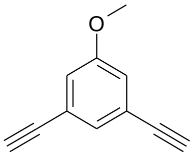

1,3-Diethynyl-5-methoxybenzene (19)[3]

To a solution of 1,3-dibromo-5-metoxybenzene (**17**) (2.66 g, 10.0 mmol) in THF/Et_3N (2:1, 30 mL) trimethylsilylacetylene (4.30 mL, 30.0 mmol), $PdCl_2(PPh_3)_2$ (0.70 g, 1.00 mmol), CuI (0.28 g, 1.50 mmol) were added and the solution was stirred under Ar at 65 °C for 20 h. The reaction mixture was cooled to room temperature, and solution of KOH in methanol (1M, 50 mL) was added. The reaction mixture was stirred for 1 h at room temperature, poured into brine (150 mL), and extracted with CH_2Cl_2 (3×50 mL), dried over Na_2SO_4, and concentrated under in vacuum. The residue was purified by column chromatography on silica gel (hexane/EtOAc 98:2) to give 1,3-diethynyl-5-methoxybenzene (**18**) (0.96 g, 61%) as light brown crystals. Mp 63–64 °C.

^{1}H NMR (400 MHz, DMSO-d_6) δ: 3.78 (s, 3H), 4.25 (s, 2H), 7.07 (d, J = 1.3 Hz, 2H), 7.12 (t, J = 1.3 Hz, 1H).

Synthesis of 2-Chloro-N-arylacetamide derivatives

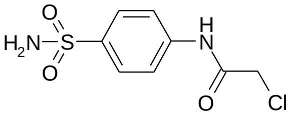

2-Chloro-*N*-(4-sulfamoylphenyl)acetamide (5a)[4]

To a solution of 4-aminobenzenesulfonamide (**4a**) (2.00 g, 11.6 mmol) in dry THF (60 mL) at 0 °C, K_2CO_3 (3.21 g, 23.2 mmol) was added. Chloroacetyl chloride (1.57 g, 13.9 mmol) was added dropwise and the mixture was stirred at 0 °C for 30 min. Water (80 mL) was added, the organic layer was separated and the aqueous layer was extracted with EtOAc (2×150 mL). The combined organic layer was washed with brine, dried over anhydrous Na_2SO_4. Solvent evaporation yielded white solid (2.82 g, 98%). Mp 214–215 °C.

^{1}H NMR (400 MHz, DMSO-d_6) δ: 4.30 (s, 2H), 7.27 (s, 2H), 7.72–7.81 (m, 4H), 10.62 (s, 1H).

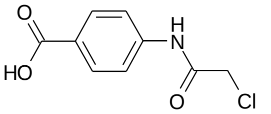

4-[(Chloroacetyl)amino]benzoic acid (5b)[5]

4-Aminobenzoic acid (**4b**) (1.00 g, 7.29 mmol) was dissolved in H_2O (100 mL) along with NaOH (0.875 g, 21.9 mmol). Chloroacetyl chloride (2.47 g, 21.9 mmol) was added dropwise at 0 °C. The mixture was stirred at 0 °C for 30 min then at room temperature overnight. The precipitate formed was collected, washed with water and dried in vacuum at 45 °C to give white solid (1.31 g, 84%). Mp 263–265 °C.

^{1}H NMR (400 MHz, DMSO-d_6) δ: 4.29 (s, 2H), 7.68–7.73 (m, 2H), 7.89–7.94 (m, 2H), 10.59 (s, 1 H), 12.72 (br s, 1 H).

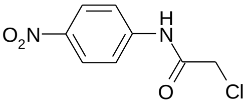

2-Chloro-*N*-(4-nitrophenyl)acetamide (5c)[6]

To a solution of 4-nitroaniline (**4c**) (1.00 g, 7.24 mmol) in dry THF (30 mL) at 0°C, K_2CO_3 (2.00 g, 14.5 mmol) was added. Chloroacetyl chloride (0.98 g, 8.69 mmol) was added dropwise at 0 °C. The mixture was stirred at 0 °C for 30 min. Water (40 mL) was added into the reaction mixture, the organic layer was separated and the aqueous layer was extracted with EtOAc (2×75

mL). The combined organic layer was washed with brine, dried over anhydrous Na_2SO_4, concentrated in vacuum to give yellow solid (1.38 g, 89%).

^{1}H NMR (400 MHz, DMSO-d_6) δ: 4.34 (s, 2H), 7.82–7.86 (m, 2H), 8.22–8.27 (m, 2H), 10.88 (s, 1H).

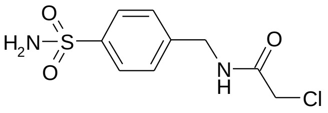

2-Chloro-N-(4-sulfamoylbenzyl)acetamide (9a)[7]

Homosulfamine (**8a**) (2.00 g, 8.98 mmol) was dissolved in 5% NaOH aqueous solution (30 mL). Chloroacetyl chloride (3.04 g, 26.9 mmol) was then added dropwise at 0 °C. The mixture was stirred at 0 °C for 30 min then at room temperature overnight. The precipitate formed was collected, washed with water and dried in vacuum at 45 °C to give white solid (0.79 g, 33%). Mp 166–167 °C.

^{1}H NMR (400 MHz, DMSO-d_6) δ: 4.14 (s, 2H), 4.37 (d, J = 6.0 Hz, 2H), 7.31 (s, 2H), 7.41–7.46 (m, 2H), 7.75–7.79 (m, 2H), 8.82 (t, J = 6.0 Hz, 1H).

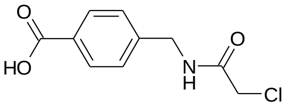

4-{[(Chloroacetyl)amino]methyl}benzoic acid (9b)

4-(Aminomethyl)benzoic acid (**9b**) (2.00 g, 13.2 mmol) was dissolved in 5% NaOH aqueous solution (30 mL). Chloroacetyl chloride (4.48 g, 39.7 mmol) was then added dropwise at 0 °C. The mixture was stirred at 0 °C for 30 min then at room temperature overnight. The precipitate formed was collected and washed with water and dried in vacuum at 45 °C to give white solid (2.09 g, 69%). Mp 214–215 °C.

IR (KBr, cm^{-1}) ν_{max}: 3337 (OH), 1673 (C=O), 1645 (C=O); ^{1}H NMR (400 MHz, DMSO-d_6) δ: 4.15 (s, 2H), 4.38 (d, J = 6.0 Hz, 2H), 7.35–7.40 (m, 2H), 7.88–7.92 (m, 2H), 8.82 (t, J = 6.0 Hz, 1H), 12.90 (br s, 1H). ^{13}C NMR (100 MHz, DMSO-d_6) δ: 42.2, 42.6, 127.3, 129.4, 129.5, 144.0, 166.2, 167.1; HRMS (ESI) $[M+H]^+$: m/z Calcd for ($C_{10}H_{11}ClNO_3$) 228.0427. Found 228.0428.

General procedure for the synthesis of 2-azido-N-arilacetamide 6 and 2-azido-N-methylarilacetamide 10 derivatives

To a solution of corresponding 2-chloro-*N*-arylacetamide **5** (1 equiv) in dry DMF (70 mL) at 0°C, NaN_3 (1.5 equiv) was added. Reaction mixture warmed to room temperature and stirred

overnight. Water (40 mL) was added into the reaction mixture and the aqueous layer was extracted with EtOAc (2×75 mL). The combined organic layers were washed with water, dried over anhydrous Na_2SO_4. The drying agent was filtered off and the filtrate concentrated.

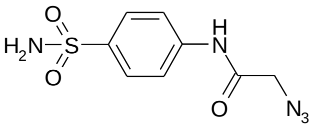

2-Azide-N-(4-sulfamoylphenyl)acetamide (6a)

Compound **6a** was prepared according to the general procedure from 2-chloro-*N*-(4-sulfamoylphenyl)acetamide (**5a**) (2.82 g, 11.3 mmol) and NaN_3 (1.11 g, 17.1 mmol) as a white solid (2.80 g, 97%). Mp 171–172 °C.

IR (KBr, cm^{-1}) ν_{max}: 3324 (NH), 3204 (NH), 2098 (N_3), 1684 (C=O), 1323 (S=O), 1154 (S=O); ^1H NMR (400 MHz, DMSO-d_6) δ: 4.09 (s, 2H), 7.27 (s, 2H), 7.72–7.80 (m, 4H), 10.47 (s, 1H). ^{13}C NMR (100 MHz, DMSO-d_6) δ: 51.3, 118.9, 126.8, 138.8, 141.3, 166.9; HRMS (ESI) $[M+H]^+$: *m/z* Calcd for ($C_8H_{10}N_5O_3S$) 256.0504. Found 256.0504.

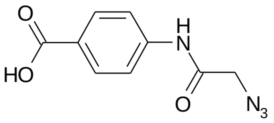

4-[(Azidoacetyl)amino]benzoic acid (6b)

Compound **6b** was prepared according to the general procedure from 4-[(chloroacetyl)amino]benzoic acid (**5b**) (0.80 g, 3.75 mmol) and NaN_3 (0.36 g, 5.61 mmol) as a white solid (0.56 g, 68%). Mp 160–161 °C.

IR (KBr, cm^{-1}) ν_{max}: 3308 (OH), 2118 (N_3), 1679 (C=O), 1608 (C=O); ^1H NMR (400 MHz, DMSO-d_6) δ: 4.09 (s, 2H), 7.68–7.73 (m, 2H), 7.89–7.93 (m, 2H), 10.43 (s, 1H), 12.73 (br s, 1H). ^{13}C NMR (100 MHz, DMSO-d_6) δ: 51.4, 118.6, 125.6, 130.5, 142.4, 166.9; HRMS (ESI) $[M+H]^+$: *m/z* Calcd for ($C_9H_9N_4O_3$) 221.0675. Found 221.0675.

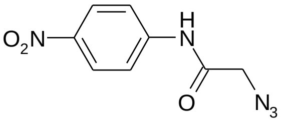

2-Azide-N-(4-nitrophenyl)acetamide (6c)

Compound **6c** was prepared according to the general procedure from 2-chloro-N-(4-nitrophenyl)acetamide (**5c**) (1.00 g, 4.66 mmol) and NaN_3 (0.454 g, 6.99 mmol) as a yellow solid (0.98 g, 95%). Mp 84–85 °C.

IR (KBr, cm^{-1}) ν_{max}: 2123 (N_3), 1674 (C=O), 1502 (N=O), 1345 (N=O); ^1H NMR (400 MHz, DMSO-d_6) δ: 4.14 (s, 2H), 7.81–7.86 (m, 2H), 8.21–8.26 (m, 2H), 10.73 (br s). ^{13}C NMR (100 MHz, DMSO-d_6) δ: 51.4, 119.0, 125.1, 142.5, 144.5, 167.4; HRMS (ESI) $[M+H]^+$: *m/z* Calcd for ($C_8H_8N_5O_3$) 222.0627. Found 222.0638.

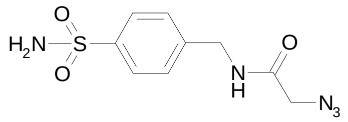

2-Azido-*N*-(sulfamoylbenzyl)acetamide (10a)

Compound **10a** was prepared according to the general procedure from 2-chloro-N-(4-sulfamoylbenzyl)acetamide **9a** (0.74 g, 2.83 mmol) and NaN_3 (0.276 g, 4.25 mmol) as a white solid (0.96 g,78%). Mp 134–135 °C.

IR (KBr, cm^{-1}) ν_{max}: 3336 (NH), 3207 (NH), 2109 (N_3), 1658 (C=O), 1312 (S=O), 1153 (S=O); ^1H NMR (400 MHz, DMSO-d_6) δ: 3.92, (s, 2H), 4.37 (d, *J* = 6.0 Hz, 2H), 7.31 (s, 2H), 7.42–7.47 (m, 2H), 7.75–7.80 (m, 2H), 8.70 (t, *J* = 6.0 Hz, 1H). ^{13}C NMR (100 MHz, DMSO-d_6) δ: 41.8, 50.8, 125.7, 127.6, 142.8, 143.0, 167.5; HRMS (ESI) $[M+H]^+$: *m/z* Calcd for ($C_9H_{12}N_5O_3S$) 270.0661. Found 270.0665.

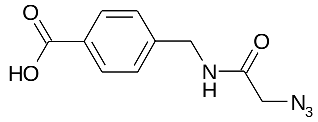

4-{[(Azidoacetyl)amino]methyl}benzoic acid (10b)

Compound **10b** was prepared according to the general procedure from 4-{[(chloroacetyl)amino]methyl}benzoic acid **9b** (2.00 g, 8.78 mmol) and NaN_3 (0.86 g, 13.18 mmol) as a white solid (1.59 g, 77%). Mp 178–179 °C.

IR (KBr, cm^{-1}) ν_{max}: 3299 (OH), 2097 (N_3), 1688 (C=O), 1645 (C=O); ^1H NMR (400 MHz, DMSO-d_6) δ: 3.93 (s, 2H), 4.37 (d, *J* = 6.0 Hz, 2H), 7.36–7.40 (m, 2H), 7.87–7.92 (m, 2H), 8.81 (t, *J* = 6.0 Hz, 1H), 12.89 (br s, 1H); ^{13}C NMR (100 MHz, DMSO-d_6) δ: 42.0, 50.8, 127.3, 129.4, 129.5, 144.1, 167.1, 167.5; HRMS (ESI) $[M+H]^+$: *m/z* Calcd for ($C_{10}H_{11}N_4O_3$) 235.0831. Found 235.0844.

General procedure for the synthesis of 1,3-bis-1,2,3-triazole derivatives

To a solution of 2-azido-*N*-arylacetamide (**6a-c** or **10a,b**; 2.5 equiv) in 5 mL (DMF/H_2O 4:1), the corresponding alkyne (**3**, **14** or **19**; 1 equiv), sodium ascorbate (1 equiv) and $CuSO_4 \cdot 5H_2O$

(0.5 equiv) was added. The resultant reaction mixture was stirred for 15 min, then acetic acid was added (5 equiv) and stirring was continued at room temperature for 1-18 hours.

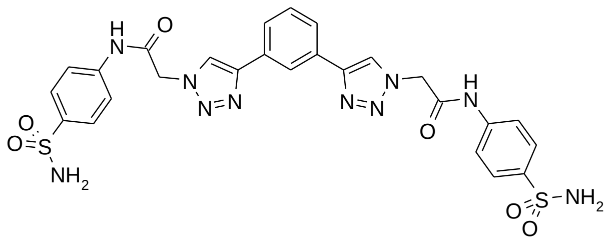

2-4-[3-(1-2-[4-(Aminosulfonyl)anilino]-2-oxoethyl-1*H*-1,2,3-triazol-4-yl)phenyl]-1*H*-1,2,3-triazol-1-yl-*N*-[4-(aminosulfonyl)phenyl]acetamide (7a)

Compound **7a** was prepared according to the general procedure from 1,3-diethynylbenzene (**3**) (0.05 g, 0.40 mmol), 2-azido-*N*-(4-sulfamoylphenyl)acetamide (**6a**) (0.25 g, 0.99 mmol), sodium ascorbate (0.08 g, 040 mmol), $CuSO_4.5H_2O$ (0.05 g. 0.20 mmol) and acetic acid (0.11 mL, 1.98 mmol). Reaction mixture was stirred at room temperature for 18 h. The solvent was evaporated under reduce pressure. The crude product was purified by reverse phase chromatography (C-18, H_2O-MeCN gradient MeCN 10-90%) to give light brown solid (0.08 g, 32%). Mp 293–294 °C.

IR (KBr, cm^{-1}) ν_{max}: 3267 (NH), 1685 (C=O), 1335 (S=O), 1157 (S=O); ^1H NMR (400 MHz, DMSO-d_6) δ: 5.47 (s, 4H), 7.28 (s, 4H), 7.56 (t, J = 7.6 Hz, 1H), 7.73–7.83 (m, 8H), 7.84–7.88 (m, 2H), 8.42 (s, 1H), 8.71 (s, 2H), 10.88 (br s, 2H); ^{13}C NMR (100 MHz, DMSO-d_6) δ: 52.5, 118.9, 121.8, 123.4, 124.6, 126.9, 129.7, 131.4, 139.0, 141.3, 146.0, 164.8; HRMS (ESI) $[M+H]^+$: *m/z* Calcd for ($C_{26}H_{25}N_{10}O_6S_2$) 637.1400. Found 637.1380.

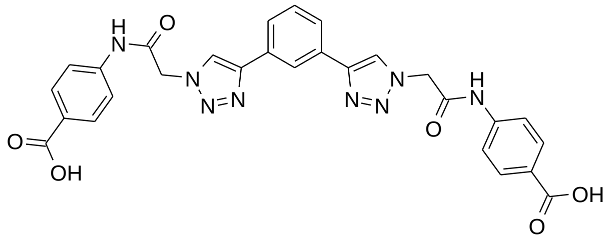

4-(2-[4-(3-1-[2-(4-Carboxyanilino)-2-oxoethyl]-1*H*-1,2,3-triazol-4-ylphenyl)-1*H*-1,2,3-triazol-1-yl]acetylamino)benzenecarboxylic acid (7b)

Compound **7b** was prepared according to the general procedure from 1,3-diethynylbenzene (**3**) (0.05 g, 0.40 mmol), 4-[(azidoacetyl)amino]benzoic acid (**6b**) (0.22 g, 0.99 mmol), sodium ascorbate (0.08 g, 0.40 mmol), $CuSO_4 \cdot 5H_2O$ (0.05 g, 0.20 mmol) and acetic acid (0.11 mL, 1.98 mmol). Reaction mixture was stirred at room temperature for 1 h, then H_2O (40 mL) was added.

Precipitate formed was filtered, washed with H_2O (2×40 mL), sat. aq. NH_4Cl (3×40 mL) and v/v 80% MeOH (2×40 mL) and air dried. It was dissolved in DMSO (40 mL) and poured into sat. aq. NH_4Cl, precipitate formed was collected, washed with H_2O (3×40 mL) and dried in vacuum to yield **7b** (0.16 g, 71%) as light yellow solid. Mp 291–292 °C.

IR (KBr, cm^{-1}) ν_{max}: 3335 (OH), 1689 (C=O), 1603 (C=O); ^{1}H NMR (300 MHz, DMSO-d_6) δ: 5.47 (s, 4H), 7.56 (t, J = 7.3 Hz, 1H), 7.67–7.79 (m, 4H), 7.86 (d, J = 7.3 Hz, 2H), 7.89–7.99 (m, 4H), 8.42 (s, 1H), 8.71 (s, 2H), 10.86 (s, 2H), 12.53 (pl s, 2H); ^{13}C NMR (100 MHz, DMSO-d_6) δ: 52.5, 118.6, 121.9, 123.4, 124.6, 126.1, 129.7, 130.5, 131.4, 142.3, 146.0, 164.8, 166.9; HRMS (ESI) $[M+H]^+$: *m/z* Calcd for ($C_{28}H_{23}N_8O_6$) 567.1741. Found 567.1710.

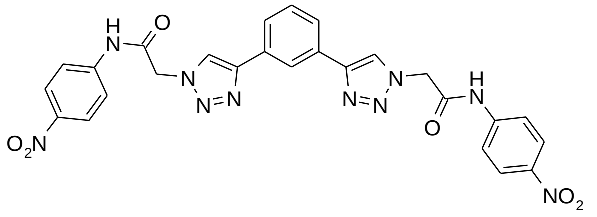

2-[4-(3-1-[2-(4-Nitroanilino)-2-oxoethyl]-1*H*-1,2,3-triazol-4-ylphenyl)-1*H*-1,2,3-triazol-1-yl]-*N*-(4-nitrophenyl)acetamide (7c)

Compound **7c** was prepared according to the general procedure from 1,3-diethynylbenzene (**3**) (0.05 g, 0.40 mmol), 2-azide-*N*-(4-nitrophenyl)acetamide (**6c**) (0.22 g, 0.99 mmol), sodium ascorbate (0.08 g, 040 mmol), $CuSO_4{\cdot}5H_2O$ (0.05 g, 0.20 mmol) and acetic acid (0.11 mL, 1.98 mmol). Reaction mixture was stirred at room temperature for 18 h. Solvent was evaporated under reduce pressure. The crude product was purified by reverse phase chromatography (C-18, H_2O-MeCN gradient MeCN 10-90%) to give light brown solid (0.09 g, 41%). Mp >307 °C dec.

IR (KBr, cm^{-1}) ν_{max}: 1695 (C=O), 1507 (N=O), 1343 (N=O); ^{1}H NMR (400 MHz, DMSO-d_6) δ: 5.51 (s, 4H), 7.56 (t, J = 7.8 Hz, 1H), 7.81–7.89 (m, 6H), 8.22–8.29 (m, 4H), 8.41–8.44 (m, 1H), 8.71 (s, 2H), 11.14 (br s, 2H); ^{13}C NMR (100 MHz, DMSO-d_6) δ: 52.5, 119.1, 121.8, 123.4, 124.7, 125.2, 129.7, 131.3, 142.7, 144.5, 146.1, 165.3; HRMS (ESI) $[M+H]^+$: *m/z* Calcd for ($C_{26}H_{21}N_{10}O_6$) 569.1646. Found 569.1684.

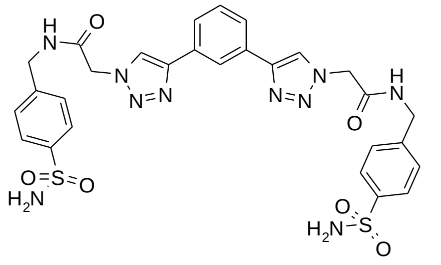

1,3-Di-{1-(*N*-1-(4-aminosulphonylbenzylamino)-2-acetyl)-1,2,3-triazol-4-yl}benzene (11a)

Compound **11a** was prepared according to the general procedure from 2-azido-*N*-(sulfamoylbenzyl)acetamide (**10a**) (0.27 g, 0.99 mmol), 1,3-diethynylbenzene (**3**) (0.05 g, 0.40 mmol), sodium ascorbate (0.08 g, 040 mmol), $CuSO_4{\cdot}5H_2O$ (0.05 g. 0.20 mmol) and acetic acid (0.11 mL, 1.98 mmol). The reaction mixture was stirred at room temperature for 18 h. The solvent was evaporated under reduced pressure. The crude product was purified by reverse phase chromatography (C-18, H_2O-MeCN gradient MeCN 10-90%) to give light brown solid (0.04 g, 17%). Mp 229-230 °C.

IR (KBr, cm^{-1}) ν_{max}: 3297 (NH), 3088 (NH), 1669 (C=O), 1329 (S=O), 1157 S=O); ^1H NMR (400 MHz, DMSO-d_6) δ: 4.42 (d, 4H, *J*=6.0 Hz), 5.27 (s, 4H), 7.32 (s, 4H), 7.46–7.51 (m, 4H), 7.54 (t, 1H, *J*=7.8 Hz), 7.76–7.81 (m, 4H), 7.83 (dd, 2H, *J*=7.8 Hz, *J*=1.6 Hz), 8.38–8.41 (m, 1H), 8.65 (s, 2H), 8.96 (t, 2H, *J*=6.0 Hz); ^{13}C NMR (100 MHz, DMSO-d_6) δ: 42.0, 51.8, 118.9, 121.7, 123.3, 124.5, 125.7, 127.7, 131.3, 142.8, 142.8, 145.9, 165.6; HRMS (ESI) $[M+H]^+$: Calcd for ($C_{28}H_{29}N_{10}O_6S_2$) 665.1713. Found 665.1730.

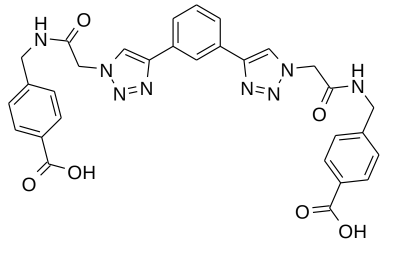

1,3-Di-{1-(*N*-1-(4-hydroxycarbonylbenzylamino)-2-acetyl)-1,2,3-triazol-4-yl}benzene (11b)

Compound **11b** was prepared according to the general procedure from 4-{[(azidoacetyl)amino]methyl}benzoic acid (**10b**) (0.32 g, 1.39 mmol), 1,3-diethynylbenzene (**3**) (0.07 g, 0.55 mmol), sodium ascorbate (0.11 g, 0.55 mmol), $CuSO_4{\cdot}5H_2O$ (0.07 g. 0.28 mmol) and acetic acid (0.16 mL, 2.77 mmol). The reaction mixture was stirred at room temperature for 1 h, then H_2O (40 mL) was added. The formed precipitate was collected, washed with H_2O (2×40 mL), sat. aq. NH_4Cl (3×40 mL) and v/v 80% MeOH (2×40 mL) and dried on air. It was dissolved in

DMSO (40 mL) and poured into sat. aq. NH_4Cl, precipitate formed was collected, washed with H_2O (3×40 mL) and dried in vacuum to obtain **11b** (0.19 g, 59%) as light brown solid. Mp 287-288 °C.

IR (KBr, cm^{-1}) ν_{max}: 3281 (OH), 1696 (C=O), 1659 (C=O); ^1H NMR (400 MHz, DMSO-*d6*) *δ*: 4.37-4.48 (m, 4H), 5.27 (s, 4H), 7.34-7.47 (m, 4H), 7.54 (t, 1H, *J*=7.6 Hz), 7.78-7.87 (m, 2H), 7.87-8.00 (m, 4H), 8.34-8.42 (m, 1H), 8.65 (s, 2H), 8.89-9.01 (m, 2H), 12.37 (pl s, 2H); ^{13}C NMR (100 MHz, DMSO-*d6*) *δ*: 42.2, 51.8, 121.8, 123.3, 124.5, 127.3, 129.4, 129.6, 130.0, 131.4, 143.7, 146.0, 165.6, 167.3; HRMS (ESI) $[M+H]^+$: *m/z* Calcd for ($C_{30}H_{27}N_8O_6$) 595.2054. Found 595.2018.

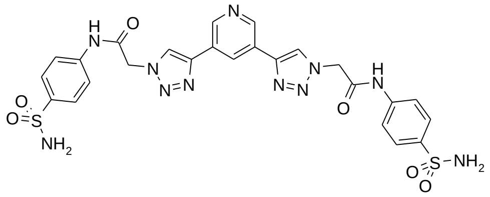

3,5-Di-{1-(*N*-1-(4-aminosulphonylphenylamino)-2-acetyl)-1,2,3-triazol-4-yl}pyridine (15a)

Compound **15a** was prepared according to the general procedure from 2-azide-*N*-(4-sulfamoylphenyl)acetamide (**6a)** (0.25 g, 0.98 mmol), 1,3-diethynylpyridine (**14**) (0.05 g, 0.39 mmol), sodium ascorbate (0.08 g, 0.39 mmol), $CuSO_4 \cdot 5H_2O$ (0.05 g, 0.20 mmol) and acetic acid (0.11 mL, 1.97 mmol). The reaction mixture was stirred at room temperature for 1 h, then H_2O (40 mL) was added. Precipitate formed was collected, washed with H_2O (2×40 mL), sat. aq. NH_4Cl (3×40 mL), v/v 80% MeOH (2×40 mL) and dried in vacuum. Compound **15a** (0.21 g, 85%) was isolated as dark brown solid. Mp 283-284 °C.

IR (KBr, cm^{-1}) ν_{max}: 3269 (NH), 3129 (NH), 1695 (C=O), 1324 (S=O), 1154 (S=O); ^1H NMR (400 MHz, DMSO-*d6*) *δ*: 5.51 (s, 4H), 7.29 (s, 4H), 7.72-7.84 (m, 8H), 8.69 (s, 2H), 8.81 (s, 1H), 8.84 (s, 2H), 10.89 (pl s, 2H); ^{13}C NMR (100 MHz, DMSO-*d6*) *δ*: 52.5, 118.9, 124.4, 124.5, 126.9, 127.6, 139.0, 141.2, 143.9, 145.4, 164.7; HRMS (ESI) $[M+H]^+$: *m/z* Calcd for ($C_{25}H_{24}N_{11}O_6S_2$) 638.1352. Found 638.1331.

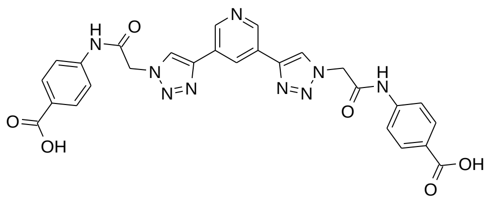

3,5-Di-{1-(*N*-1-(4-hydroxycarbonylphenylamino)-2-acetyl)-1,2,3-triazol-4-yl}pyridine (15b)

Compound **15b** was prepared according to the general procedure from 4-[(azidoacetyl)amino]benzoic acid (**6b**) (0.22 g, 0.98 mmol), 1,3-diethynylpyridine (**14**) (0.05 g, 0.39 mmol), sodium ascorbate (0.08 g, 0.39 mmol), $CuSO_4{\cdot}5H_2O$ (0.05 g, 0.20 mmol) and acetic acid (0.11 mL, 1.97 mmol). Reaction mixture was stirred at room temperature for 1 h, then H_2O (40 mL) was added. Precipitate formed was collected, washed with H_2O (2×40 mL), sat. aq. NH_4Cl (3×40 mL) and v/v 80% MeOH (2×40 mL) and dried on air. Then it was dissolved in DMSO (40 mL) and poured into sat. aq. NH_4Cl, precipitate formed was collected, washed with H_2O (3×40 mL) and dried in vacuum. Compound **15b** (0.09 g, 39%) was isolated as light brown solid. Mp >260 °C dec.

IR (KBr, cm^{-1}) ν_{max}: 3288 (OH), 1684 (C=O), 1603 (C=O); ^1H NMR (400 MHz, DMSO-d_6) δ: 5.50 (s, 4H), 7.67-7.80 (m, 4H), 7.84-8.02 (m, 4H), 8.68-8.79 (m, 2H), 8.80-8.90 (m, 2H), 9.11 (pl s, 1H), 10.85 (s, 2H), 12.73 (pl s, 2H); ^{13}C NMR (100 MHz, DMSO-d_6) δ: 52.6, 118.6, 124.2, 126.0, 126.9, 128.5, 130.6, 142.3, 143.3, 145.6, 164.7, 166.9; HRMS (ESI) $[M+H]^+$: *m/z* Calcd for ($C_{27}H_{22}N_9O_6$) 568.1693. Found 568.1671.

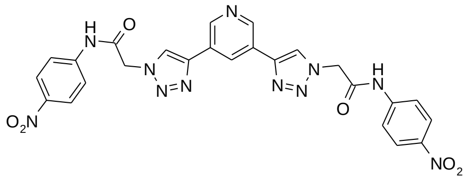

3,5-Di-{1-(*N*-1-(4-nitrophenylamino)-2-acetyl)-1,2,3-triazol-4-yl}pyridine (15c)

Compound **15c** was prepared according to the general procedure from 2-azide-*N*-(4-nitrophenyl)acetamide (**6c**) (0.22 g, 0.98 mmol), 1,3-diethynylpyridine **14** (0.05 g, 0,39 mmol), sodium ascorbate (0.08 g, 0.39 mmol), $CuSO_4{\cdot}5H_2O$ (0.05 g, 0.20 mmol) and acetic acid (0.11 mL, 1.97 mmol). The reaction mixture was stirred at room temperature for 1 h, then H_2O (40 mL) was added. Precipitate formed was collected, washed with H_2O (2×40 mL), sat. aq. NH_4Cl (3×40 mL), v/v 80% MeOH (2×40 mL) and dried on air. Then it was dissolved in DMSO (40 mL) and poured into sat. aq. NH_4Cl. Precipitate formed was collected, washed with H_2O (3×40 mL) and dried in vacuum. Compound **15c** (0.16 g, 73%) was isolated as light yellow solid. Mp >282 °C dec.

IR (KBr, cm^{-1}) ν_{max}: 1706 (C=O), 1507 (N=O), 1348 (N=O); ^1H NMR (400 MHz, DMSO-*d6*) *δ*: 5.54 (s, 4H), 7.79-7.92 (m, 4H), 8.20-8.33 (m, 4H), 8.74 (s, 1H), 8.85 (s, 2H), 9.09 (pl s, 2H), 11.14 (s, 2H); ^{13}C NMR (100 MHz, DMSO-*d6*) *δ*: 52.6, 119.1, 124.2, 125.2, 128.4, 142.7, 143.3, 144.5, 145.7, 165.2; HRMS (ESI) $[M+H]^+$: *m/z* Calcd for ($C_{25}H_{20}N_{11}O_6$) 570.1598. Found 570.1572.

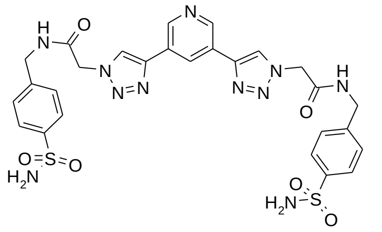

3,5-Di-{1-(*N*-1-(4-aminosulphonylbenzylamino)-2-acetyl)-1,2,3-triazol-4-yl}pyridine (16a)

Compound **16a** was prepared according to the general procedure from 2-azido-*N*-(sulfamoylbenzyl)acetamide (**10a**) (0.27 g, 0.98 mmol), 1,3-diethynylpyridine (**14**) (0.05 g, 0.39 mmol), sodium ascorbate (0.08 g, 0.39 mmol), $CuSO_4 \cdot 5H_2O$ (0.05 g, 0.20 mmol) and acetic acid (0.11 mL, 1.97 mmol). The reaction mixture was stirred at room temperature for 3 h, then H_2O (40 mL) was added. Precipitate formed was collected, washed with H_2O (2×40 mL), sat. aq. NH_4Cl (3×40 mL), v/v 80% MeOH (2×40 mL) and dried on air. Then it was dissolved in DMSO (40 mL) and poured into sat. aq. NH_4Cl. Precipitate formed was collected, washed with H_2O (3×40 mL) and dried in vacuum. Compound **16a** (0.16 g, 63%) was isolated as light yellow solid. Mp 252-253 °C.

IR (KBr, cm^{-1}) ν_{max}: 3295 (NH), 3050 (NH), 1669 (C=O), 1330 (S=O), 1157 (S=O); ^1H NMR (400 MHz, DMSO-*d6*) *δ*: 4.35-4.49 (m, 4H), 5.31 (s, 4H), 7.33 (s, 4H), 7.42-7.55 (m, 4H), 7.73-7.85 (m, 4H), 8.67-8.74 (m, 1H), 8.76-8.83 (m, 2H), 8.93-9.02 (m, 2H), 9.04 (s, 2H); ^{13}C NMR (100 MHz, DMSO-*d6*) *δ*: 42.1, 51.9, 124.1, 125.7, 126.9, 127.7, 128.4, 142.8, 143.2, 145.5, 165.6; HRMS (ESI) $[M+H]^+$: *m/z* Calcd for ($C_{27}H_{28}N_{11}O_6S_2$) 666.1665. Found 666.1640.

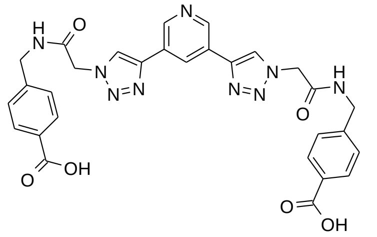

3,5-Di-{1-(*N*-1-(4-hydroxycarbonylbenzylamino)-2-acetyl)-1,2,3-triazol-4-yl}pyridine (16b)

Compound **16b** was prepared according to the general procedure from 4-{[(azidoacetyl)amino]methyl}benzoic acid (**10b**) (0.23 g, 0.98 mmol), 1,3-diethynylpyridine (**14**) (0.05 g, 0.39 mmol), sodium ascorbate (0.08 g, 0.39 mmol), $CuSO_4 \cdot 5H_2O$ (0.05 g, 0.20 mmol) and acetic acid (0.11 mL, 1.97 mmol). Reaction mixture was stirred at room temperature for 3 h, then H_2O (40 mL) was added. Precipitate formed was collected, washed with H_2O (2×40 mL), sat. aq. NH_4Cl (3×40 mL), v/v 80% MeOH (2×40 mL) and dried on air. Then it was dissolved in DMSO (40 mL) and poured into sat. aq. NH_4Cl. Precipitate formed was collected, washed with H_2O (3×40 mL) and dried in vacuum. Compound **16b** (0.11 g, 46%) was isolated as light brown solid. Mp 273-274 °C.

IR (KBr, cm^{-1}) ν_{max}: 3282 (OH), 1688 (C=O), 1653 (C=O); ^1H NMR (400 MHz, DMSO-d_6) δ: 4.43 (d, 4H, J=5.7 Hz), 5.31 (s, 4H), 7.38-7.44 (m, 4H), 7.88-7.95 (m, 4H), 8.69-8.72 (m, 1H), 8.77-8.81 (m, 2H), 9.00 (t, 2H, J=5.7 Hz), 9.06 (s, 2H), 12.85 (pl s, 2H); ^{13}C NMR (100 MHz, DMSO-d_6) δ: 42.2, 51.9, 124.1, 126.9, 127.3, 128.4, 129.4, 130.3, 143.2, 143.5, 145.5, 165.6, 167.3; HRMS (ESI) $[M+H]^+$: *m/z* Calcd for ($C_{29}H_{26}N_9O_6$) 596.2006. Found 596.1978.

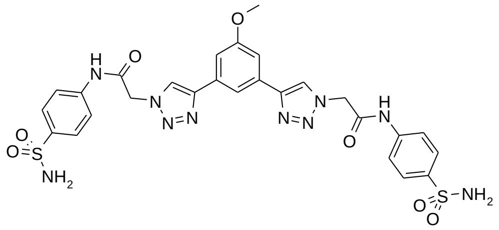

3,5-Di-{1-(*N*-1-(4-aminosulphonylphenylamino)-2-acetyl)-1,2,3-triazol-4-yl}anisole (20a)

Compound **20a** was prepared according to the general procedure from 2-azide-*N*-(4-sulfamoylphenyl)acetamide (**6a**) (0.20 g, 0.80 mmol), 1,3-diethynyl-5-methoxybenzene (**19**) (0.05 g, 0.32 mmol), sodium ascorbate (0.06 g, 0.32 mmol), $CuSO_4 \cdot 5H_2O$ (0.05 g, 0.16 mmol) and acetic acid (0.09 mL, 1.60 mmol). The reaction mixture was stirred at room temperature for 3 h, then H_2O (40 mL) was added. Precipitate formed was collected, washed with H_2O (2×40 mL), sat. aq. NH_4Cl (3×40 mL), v/v 80% MeOH (2×40 mL) and dried on air. Then it was dissolved in DMSO (40 mL)

and poured into sat. aq. NH_4Cl. Precipitate formed was collected, washed with H_2O (3×40 mL) and dried in vacuum. Compound **20a** (0.14 g, 64%) was isolated as light grey solid. Mp 285-286 °C.

IR (KBr, cm^{-1}) ν_{max}: 3273 (NH), 3056 (NH), 1689 (C=O), 1319 (S=O), 1156 (S=O); ^1H NMR (400 MHz, DMSO-d_6) δ: 3.90 (s, 3H), 5.47 (s, 4H), 7.28 (s, 4H), 7.39-7.47 (m, 2H), 7.70-7.87 (m, 8H), 7.99-8.07 (m, 1H), 8.73 (s, 2H), 10.92 (s, 2H); ^{13}C NMR (100 MHz, DMSO-d_6) δ: 52.5, 55.4, 110.1, 114.5, 118.9, 123.6, 126.9, 132.6, 139.0, 141.3, 146.0, 160.2, 164.8; HRMS (ESI) $[M+H]^+$: *m/z* Calcd for ($C_{27}H_{27}N_{10}O_7S_2$) 667.1506. Found 667.1477.

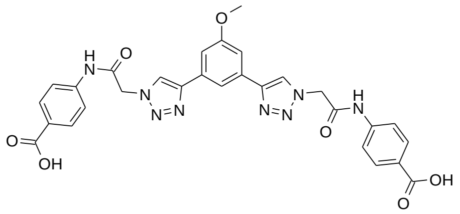

3,5-Di-{1-(*N*-1-(4-hydroxycarbonylphenylamino)-2-acetyl)-1,2,3-triazol-4-yl}anisole (20b)

Compound **20b** was prepared from 4-[(azidoacetyl)amino]benzoic acid (**6b**) (0.18 g, 0.80 mmol), 1,3-diethynyl-5-methoxybenzene (**19**) (0.05 g, 0.32 mmol), sodium ascorbate (0.06 g, 0.32 mmol), $CuSO_4 \cdot 5H_2O$ (0.05 g, 0.16 mmol) and acetic acid (0.09 mL, 1.60 mmol). The reaction mixture was stirred at room temperature for 3 h, then H_2O (40 mL) was added. Precipitate formed was collected, washed with H_2O (2×40 mL), sat. aq. NH_4Cl (3×40 mL), v/v 80% MeOH (2×40 mL) and dried on air. Then it was dissolved in DMSO (40 mL) and poured into sat. aq. NH_4Cl. Precipitate formed was collected, washed with H_2O (3×40 mL) and dried in vacuum. Compound **20b** (0.11 g, 56%) was isolated as light brown solid. Mp 285-286 °C.

IR (KBr, cm^{-1}) ν_{max}: 3291 (OH), 1684 (C=O), 1603 (C=O); ^1H NMR (400 MHz, DMSO-d_6) δ: 3.90 (s, 3H), 5.46 (s, 4H), 7.39-7.45 (m, 2H), 7.68-7.76 (m, 4H), 7.89-7.97 (m, 4H), 8.00-8.04 (m, 1H), 8.73 (s, 2H), 10.87 (s, 2H), 12.58 (pl s, 2H); ^{13}C NMR (100 MHz, DMSO-d_6) δ: 52.5, 55.4, 110.0, 114.5, 118.6, 123.6, 126.2, 130.6, 132.6, 142.3, 146.0, 160.2, 164.7, 167.0; HRMS (ESI) $[M+H]^+$: *m/z* Calcd for ($C_{29}H_{25}N_8O_7$) 597.1846. Found 597.1820.

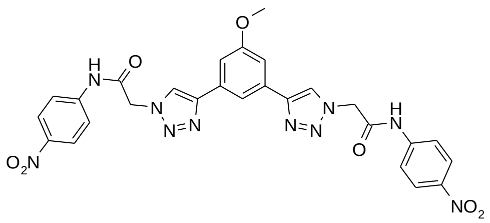

3,5-Di-{1-(*N*-1-(4-nitrophenylamino)-2-acetyl)-1,2,3-triazol-4-yl}anisole (20c)

Compound **20c** was prepared according to the general procedure from 2-azide-*N*-(4-nitrophenyl)acetamide (**6c**) (0.18 g, 0.80 mmol), 1,3-diethynyl-5-metoxybenzene (**19**) (0.05 g, 0.32 mmol), sodium ascorbate (0.06 g, 0.32 mmol), $CuSO_4{\cdot}5H_2O$ (0.05 g, 0.16 mmol) and acetic acid (0.09 mL, 1.60 mmol). The reaction mixture was stirred at room temperature for 3 h, then H_2O (40 mL) was added. Precipitate formed was filtered, washed with H_2O (2×40 mL), sat. aq. NH_4Cl (3×40 mL), v/v 80% MeOH (2×40 mL) and dried on air. The it was dissolved in DMSO (40 mL) and poured into sat. aq. NH_4Cl. Precipitate formed was collected, washed with H_2O (3×40 mL) and dried in vacuum. Compound **20c** (0.09 g, 47%) was isolated as light brown solid. Mp 262-263 °C.

IR (KBr, cm^{-1}) ν_{max}: 1696 (C=O), 1507 (N=O), 1343 (N=O); ^{1}H NMR (400 MHz, DMSO-*d*$_6$) δ: 3.90 (s, 3H), 5.51 (s, 4H), 7.36-7.49 (m, 2H), 7.75-7.96 (m, 4H), 7.97-8.09 (m, 1H), 8.17-8.37 (m, 4H), 8.73 (s, 2H), 11.15 (s, 2H); ^{13}C NMR (100 MHz, DMSO-*d*$_6$, δ): 52.5, 55.4, 110.0, 114.4, 119.1, 123.6, 125.1, 132.6, 142.6, 144.5, 146.0, 160.2, 165.3; HRMS (ESI) $[M+H]^+$: *m/z* Calcd for ($C_{27}H_{23}N_{10}O_7$) 599.1751. Found 599.1727.

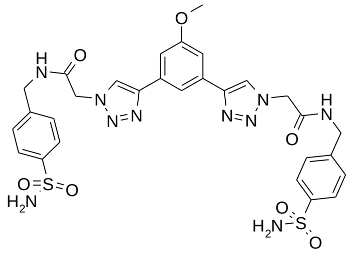

3,5-Di-{1-(*N*-1-(4-aminosulphonylbenzylamino)-2-acetyl)-1,2,3-triazol-4-yl}anisole (21a)

Compound **21a** was prepared according to the general procedure from 2-azido-*N*-(sulfamoylbenzyl)acetamide (**10a**) (0.22 g, 0.80 mmol), 1,3-diethynyl-5-metoxybenzene (**19**) (0.05 g, 0.32 mmol), sodium ascorbate (0.06 g, 0.32 mmol), $CuSO_4{\cdot}5H_2O$ (0.05 g, 0.16 mmol) and acetic acid (0.09 mL, 1.60 mmol). The reaction mixture was stirred at room temperature for 3 h, then H_2O (40 mL) was added. Precipitate formed was collected, washed with H_2O (2×40 mL), sat. aq. NH_4Cl (3×40 mL), v/v 80% MeOH (2×40 mL) and dried on air. Then it was dissolved in DMSO (40 mL)

and poured into sat. aq. NH_4Cl. Precipitate formed was collected, washed with H_2O (3×40 mL) and dried in vacuum. Compound **21a** (0.07 g, 31%) was isolated as light brown solid. Mp 225-226 °C.

IR (KBr, cm^{-1}) ν_{max}: 3292 (NH), 3093 (NH), 1668 (C=O), 1331 (S=O), 1158 (S=O); ^1H NMR (400 MHz, DMSO-d_6) δ: 3.88 (s, 3H), 4.42 (d, 4H, J=6.0 Hz), 5.26 (s, 4H), 7.32 (s, 4H), 7.40 (d, 2H, J=1.2 Hz), 7.46-7.51 (m, 4H), 7.77-7.82 (m, 4H), 8.00 (t, 1H, J=1.2 Hz), 8.67 (s, 2H), 8.96 (t, 2H, J=6.0 Hz); ^{13}C NMR (100 MHz, DMSO-d_6) δ: 42.0, 51.8, 55.3, 110.0, 114.4, 123.5, 125.7, 127.7, 132.6, 142.8, 142.8, 145.9, 160.2, 165.6; HRMS (ESI) $[M+H]^+$: m/z Calcd for ($C_{29}H_{31}N_{10}O_7S_2$) 695.1819. Found 695.1791.

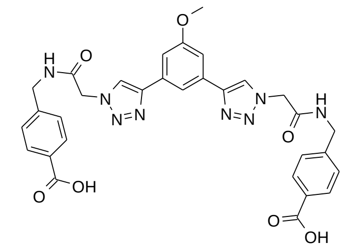

3,5-Di-{1-(*N*-1-(4-hydroxycarbonylbenzylamino)-2-acetyl)-1,2,3-triazol-4-yl}anisole (21b)

Compound **21b** was prepared according to the general procedure from 4-{[(azidoacetyl)amino]methyl}benzoic acid (**10b**) (0.19 g, 0.80 mmol), 1,3-diethynyl-5-metoxybenzene (**19**) (0.05 g, 0,32 mmol), sodium ascorbate (0.06 g, 0.32 mmol), $CuSO_4 \cdot 5H_2O$ (0.05 g, 0.16 mmol) and acetic acid (0.09 mL, 1.60 mmol). Reaction mixture was stirred at room temperature for 3 h, then H_2O (40 mL) was added. Precipitate formed was filtered, washed with H_2O (2×40 mL), sat. aq. NH_4Cl (3×40 mL), v/v 80% MeOH (2×40 mL) and dried on air. Then it was dissolved in DMSO (40 mL) and poured into sat. aq. NH_4Cl. Precipitate formed was collected, washed with H_2O (3×40 mL) and dried in vacuum. Compound **21b** (0.17 g, 85%) was isolated as light brown solid. Mp 276-277 °C.

IR (KBr, cm^{-1}) ν_{max}: 3290 (OH), 1684 (C=O), 1611 (C=O); ^1H NMR (400 MHz, DMSO-$d_6$$\delta$: 3.89 (s, 3H), 4.32-4.51 (m, 4H), 5.27 (s, 4H), 7.32-7.51 (m, 6H), 7.85-7.97 (m, 4H), 7.97-8.05 (m, 1H), 8.68 (s, 2H), 8.91-9.02 (m, 2H), 12.49 (pl s, 2H); ^{13}C NMR (100 MHz, DMSO-d_6) δ: 42.2, 51.8, 55.4, 110.0, 114.4, 123.5, 127.4, 129.4, 129.7, 132.6, 143.8, 145.9, 160.2, 165.6, 167.2; HRMS (ESI) $[M+H]^+$: m/z Calcd for ($C_{31}H_{29}N_8O_7$) 625.2159. Found 625.2129.

LDHA inhibition assay

Lactate Dehydrogenase Assay Kit (Abcam, ab102526) and recombinant human LDHA protein (Abcam, ab93699) were used to assess the inhibitory effect of the tested compounds. First we generated a NADH standard curve for colorimetric detection by measuring the OD (450 nm) at different molar amounts of NADH. The linear regression equation of the curve was derivatized in Graphpad Prism. Tested compounds were dissolved in DMSO and their inhibitory activity was assessed at 140 and 300 μM strength. Galloflavin (Sigma, SML0776) at the same molar concentration was used as a known LDH inhibitor. The master reaction mix composed of LDH assay buffer and 10 ng human LDHA substrate per reaction was prepared. Tested compounds were added and the final reaction volume was adjusted to 50 µL. Absorbance at 450 nm was taken after 2-3 minutes (T-initial) and continued every minute for 118 mins (T-final). The change in measurement over time (delta A450) was calculated as T-final minus T-initial. The amount of NADH generated by the assay between T-initial and T-final was deduced by comparing the A450 of each sample to the standard NADH standard curve. LDHA activity was calculated in milliunits per mL as the amount of NADH generated by the assay / (reaction time in minutes (118) × reaction volume in mL (0.005).

References

1. Neenan TX, Whitesides GM. Synthesis of High Carbon Materials from Acetylenic Precursors. Preparation of Aromatic Monomers Bearing Multiple Ethynyl Groups. J Org Chem. 1988; 53: 2489-2496.
2. Goto, H.; Heemstra, J. M.; Hill, D. J.; Moore, J. S. Single-Site Modifcation and Their Effect on the Folding Stability of *m*-Phenylene Ethynylene Oligomers. Org Lett. 2004; 6: 889-892.
3. Beves JE, Blanco V, Blight BA, Carrillo R, D'Souza DM, Howgego D, Leigh DA, Slawin AMZ, Symes MD. Towards Metal Complexes that can Directionally Walk Along Tracks: Controlled Stepping of a Molecular Biped with a Palladium(II) Foot. J Am Chem Soc. 2014; 136: 2094-2100.
4. Addy PS, Saha B, Singh PND, Das AK, Bush JT, Lejeune C, Schofield CJ, Basak A. 1,3,5-Trisubstituted benzenes as fluorescent photoaffinity probes for human carbonic anhydrase II capture. Chem Commun. 2013; 49: 1930-1932.

5. Harte, AJ, Gunnlaugsson T. Synthesis of α-chloroamides in water. Tetrahedron Lett. 2006; 47: 6321-6324.
6. Deepkumar J, Kalpesh P. Synthesis and evaluation of novel benzimidazole derivatives as antimicrobial agents. Med Chem Res. 2014; 23: 1290-1299.
7. Addy PS. Saha B. Panja A. Das AK. Basak A. Design and synthesis of azobenzene template based sulfonamide for capture of HCAII: dependence of efficiency on *E-Z* geometry. Tetrahedron Lett. 2014; 55: 2625-2628.
